# Mapping Quantitative Trait Loci (QTLs) for Hundred-Pod and Hundred-Seed Weight under Seven Environments in a Recombinant Inbred Line Population of Cultivated Peanut (*Arachis hypogaea* L.)

**DOI:** 10.3390/genes14091792

**Published:** 2023-09-13

**Authors:** Penghui Miao, Xinhao Meng, Zeren Li, Sainan Sun, Charles Y. Chen, Xinlei Yang

**Affiliations:** 1 State Key Laboratory of North China for Crop Improvement and Regulation, North China Key Laboratory for Crop Germplasm Resources of Education Ministry, Key Laboratory of Crop Germplasm Resources of Hebei Province, Hebei Agricultural University, Baoding 071001, China; 2 Department of Crop, Soil and Environmental Sciences, Auburn University, Auburn, AL 36849, USA

**Keywords:** cultivated peanut, HPW, HSW, integrated genetic map, QTL mapping

## Abstract

The cultivated peanut (*Arachis hypogaea* L.) is a significant oil and cash crop globally. Hundred-pod and -seed weight are important components for peanut yield. To unravel the genetic basis of hundred-pod weight (HPW) and hundred-seed weight (HSW), in the current study, a recombinant inbred line (RIL) population with 188 individuals was developed from a cross between JH5 (JH5, large pod and seed weight) and M130 (small pod and seed weight), and was utilized to identify QTLs for HPW and HSW. An integrated genetic linkage map was constructed by using SSR, AhTE, SRAP, TRAP and SNP markers. This map consisted of 3130 genetic markers, which were assigned to 20 chromosomes, and covered 1998.95 cM with an average distance 0.64 cM. On this basis, 31 QTLs for HPW and HSW were located on seven chromosomes, with each QTL accounting for 3.7–10.8% of phenotypic variance explained (PVE). Among these, seven QTLs were detected under multiple environments, and two major QTLs were found on B04 and B08. Notably, a QTL hotspot on chromosome A08 contained seven QTLs over a 2.74 cM genetic interval with an 0.36 Mb physical map, including 18 candidate genes. Of these, *Arahy.D52S1Z*, *Arahy.IBM9RL*, *Arahy.W18Y25*, *Arahy.CPLC2W* and *Arahy.14EF4H* might play a role in modulating peanut pod and seed weight. These findings could facilitate further research into the genetic mechanisms influencing pod and seed weight in cultivated peanut.

## 1. Introduction

The cultivated peanut (*Arachis hypogaea* L.), an allotetraploid (2n = 4x = 40) crop, is an important oil crop worldwide [1]. Global annual peanut production is on a rapid upward trend, from 2015 (45 million tons) to 2021 (53.9 million tons) (FAO, 2021), but still cannot satisfy the demand of the growing global population. Increasing peanut yield per unit area remains a tremendous challenge for peanut breeders. Various agronomic characteristics, including total branch numbers (TBNs), lateral branch angle (LBA) and the size of the pod and seed, affect the yield of peanuts [2,3,4,5]. HPW and HSW, which are mainly determined by pod and seed weight and size, are vital components. They are typical quantitative traits, but their underlying genetic basis is yet to be thoroughly researched [6]. By creating a HDGM and identifying QTLs for HPW and HSW, along with mining molecular markers closely associated with yield traits, a theoretical groundwork can be laid for further revealing the genetic basis of yield traits, enhancing peanut production.

Up to the present date, various molecular markers have been created to develop genetic linkage maps in the peanut, such as RAPD, RFLP and SSR [7,8,9,10,11,12,13,14,15]. Since the peanut is an interspecific hybrid, the narrow genetic diversity has resulted in a comparatively low density of the linkage map in previous studies [16]. With the completion of the peanut genome sequencing, a large number of single nucleotide polymorphisms (SNPs) were successfully discovered to constitutionally enable genetic mapping with sufficient density [17,18,19,20,21,22]. In recent years, some HDGMs have been developed using SNP markers in both the diploid and allotetraploid peanut genome [5,23,24]. Li et al. constructed a SNP-based HDGM including 2808 SNPs covering 1308.2 cM [25]. The other HDGMs were constructed, which included 2334 markers with 68 SSRs and 2266 SNPs [26] and 2996 SNPs and 330 SSRs [27]. HDGM provides crucial information for the precise extraction of QTL linked with interest traits [14,28].

Quantitative trait loci for yield-related characteristics were identified using segregated populations in peanuts, such as PL (pod length), PW (pod width), SL (seed length), SW (seed width), HPW (hundred-pod weight) and HSW (hundred-seed weight) [24,29,30,31,32]. Nevertheless, as the two key investigation traits of pod and seed weight and size, the majority of QTLs for the two traits showed a small effect, with a phenotypic variation explained (PVE) result of less than 10%. Up to now, Luo et al. [33] have employed a RIL population (Yuanza 9102 × Xuzhou 68-4), and identified three major effective QTLs affecting HPW across four seasons. Wang et al. [23] established a RIL (ZH16 × sd-H1) and obtained two QTLs for HPW (5.86–14.46% of PVE) and six QTLs for HSW (5.17–17.95% of PVE) under three environments. Mondal et al. [34] utilized a RIL population (VG 9514 × TAG 24), leading to the identification of nine QTLs for HSW spread over six environments, which accounted for 6.71–23.88% of PVE. Kunta et al. [24] developed a RIL (Hanoch × Harariused) and discovered 30 QTLs across two seasons, including eight QTLs for 50-pod weight and -seed weight. Of these, three exhibited main effects, explaining 6.2 to 13.9% of PVE. Similarly, Gangurde et al. [35] also used a RIL population (Chico × ICGV02251) and detected seven QTLs associated with HSW in three years, responsible for 6.69–21.29% of PVE. Guo et al. [36], using a RIL derived from Zhonghua 5 and ICGV 86699, identified 15 QTLs for HSW across six settings, explaining a phenotypic variation comprising 4.08–17.89%. Within these QTLs, only three displayed main effects. Nevertheless, most QTLs cannot be detected repeatedly in multiple environments and the number of QTLs showing stable expression is still relatively low. Stable QTLs are those that have been consistently detected across multiple years in multiple environments. Obviously, there is still a lack of main-effect QTLs for stable expression in multiple environments.

Stable expression of QTLs in multiple environments is important for revealing the genetic mechanisms of crop growth and development. In our current research, to further elucidate the candidate regions of the genomic impact of pod and seed weight in peanuts, we developed a RIL population of 188 families, achieved by crossing two cultivated peanut species. “Jihua5 (JH5)”, as the female receptor, was a large-seed and erect plant type, and “M130,”as the male donor, was a small-seed with spreading plant type. HPW and HSW were significantly different between the two parents, and they presented plentiful variations in RIL generation; thus, they were suitable for QTL localization. Here, we gathered genotype data of SSR, AhTE, SRAP, TRAP and SNP markers to construct a novel HDGM. To test the practicability of the map, QTLs for the HPW and HSW were mapped across seven environments over four years. Interestingly, a QTL hotspot was discovered on chromosome A08, which holds potential significance for the future breeding of peanut pod and seed traits.

## 2. Materials and Methods

### 2.1. Plant Materials and Multiple Environment Trials

**Figure 1:**
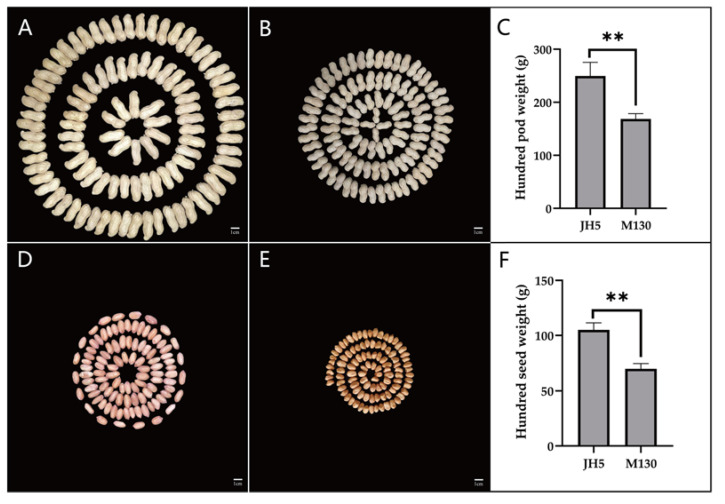
Phenotypic features of pod and seed weight of both JH5 and M130. (**A**) HPW of JH5. (**B**) HPW of M130. (**C**) *t* test for HPW of JH5 and M130. (**D**) HSW of JH5. (**E**) HSW of M130. (**F**) *t* test for HSW of JH5 and M130. Scale bar was 1 cm. ** showed significant differences at the levels of 0.01.

A RIL population was established through the F_8:11_ generation from a cross between “JH5” and “M130” using the single seed descent (SSD) method, a high-density genetic linkage map was constructed, and QTL analysis was performed for both HPW and HSW. JH5, as a female parent, was a peanut cultivar with large pods and seeds. M130, as a male parent, was a peanut germplasm with small pods and seeds (Figure 1). The 188 RILs and their parents were planted under seven environments over four years, including the Qingyuan experimental field (QY, N38°40′ and E115°30′) in the years 2017, 2018 and 2020, Daming (DM, N35°57′and E115°09ʹ) in the years 2017 and 2018, Qian’an (QA, N39°99′ and E118°70′) in 2018, and Xinle (XL, N38°15′ and E114°30′) in 2019, which were referred to as 17QY, 17DM, 18QY, 18DM, 18QA, 19XL and 20QY, respectively. We applied a randomized block design to the 188 lines with two replications, and crop field management followed local requirements. Each plot, with 10 plants, was grown in one row, the row length, row spacing and planted spacing of each one was 1.8 m, 0.5 m and 0.2 m, respectively. The parental lines were planted after every 20 rows as controls. Planting of seeds took place in May and harvest occurred in September for each experiment.

### 2.2. Traits Measurement and Statistical Analysis

Eight typical plants from each plot were harvested and picked ripe and plum-pod at the mature stage. HPW and HSW were evaluated utilizing an electronic balance with three replicates for accurate measurements. Data analysis was conducted using Prism 8.0 (GraphPad software), which assisted in analyzing descriptive statistics and variance components. This software was also vital in deducing the Pearson’s correlation coefficient amidst HPW and HSW. To validate whether the data for the two traits conformed to a normal distribution, we employed the Shapiro–Wilk test (*w*-test) for the normality evaluation of the phenotypic data. The broad-sense heritability (*h*_B_^2^) for HPW and HSW across seven environments was quantified via: *h*_B_^2^ = *σ_g_^2^*/(*σ_g_^2^*+*σ_ge_^2^*/*n*+*σ_e_^2^*/*nr*), where *σ_g_^2^*, *σ_ge_^2^* and *σ_e_^2^* symbolize the genetic variance component, genotype–environment interaction (G × E) variance component, and the random error variance component, respectively. Herein, ‘*n*’ signifies the number of environments and ‘*r*’ denotes the number of replications encompassed in each field experiment.

### 2.3. Marker Polymorphism and Analysis

Total genomic DNA was extracted from fresh leaves of RILs and two parents following the method of Wang et al. [23]. A sum of 8091 markers was obtained to screen the polymorphism of the two parents. Among these: 2808 polymorphic SNP markers from our previous research [25], 3964 pairs of SSR and 926 transposon element markers (AhTE) (https://legacy.peanutbase.org/, accessed on 15 August 2019), 238 pairs of SRAP primers [37] and 155 pairs of TRAP primers [38]. Primers were synthesized by Genewiz (Suzhou, China). The polymerase chain reaction (PCR) system of SSR and AhTE was conducted in a 10 μL mixture, including 5 μL of 2 × Es *Taq* Master Mix (Cwbio, Taizhou, China), 1 Μl of template DNA (10 ng/Μl), 0.5 Μl of forward and reverse primer (10 Μm/Μl), and 2 Μl double-distilled water. The PCR procedure involved the following steps: 95 °C for 5 min, then 30 cycles of 94 °C/40 s, 55 °C/40 s and 72 °C/60 s, final extension at 72 °C for 10 min and a cool-down process at 4 °C. In addition, SRAP and TRAP PCR amplification procedures were performed as described in Li and Quiro [37] and Hu and Vick [38]. The PCR products were investigated using 8% non-denaturing polyacrylamide gels. Silver staining was performed as described by Yang et al. [39]. Subsequently, the polymorphic markers were deployed to screen the RIL population.

### 2.4. Construction of Integrated Genetic Linkage Map

Combining SSR, AhTE, SRAP, TRAP in our current study and the previously reported SNP marker [25], an integrated genetic linkage map was constructed using JoinMap^®^ 4 [40] with a logarithm of odds (LOD) threshold of 3.0 and a maximal distance of 50 cM. The identification of segregation distortion loci was achieved by using the chi-square (χ^2^) test, and the construction of the genetic map excluded any molecular markers deviating from the expected 1:1 ratio. Genetic map distances were calculated by the Kosambi function [41], with a recombination frequency of 0.45. The genetic linkage groups were graphically presented using Mapchart 2.32 [42].

### 2.5. QTL Identification and Candidate Genes Prediction for QTL Hotspot

QTL IciMapping V4.2 [43] (statistical model: ICIM-ADD) was employed to identify QTLs for HPW and HSW. For each trait, a walk step of 0.5 cM and LOD threshold were estimated by permutation test 1000 times to determine a significant QTL. The QTL nomenclature was adopted according to Tanksley and McCouch [44]. A major QTL has more than 10% phenotypic variation explained (PVE) [34]. QTLs in the same location or overlapping region on the same chromosome are defined as a QTL hotspot. Subsequently, the candidate genes of the QTL hotspot were found according to the physical position on the reference genome of flanking markers. Then, candidate genes were analyzed for GO and KEGG enrichment.

## 3. Results

### 3.1. Phenotypic Analysis

**Figure 2:**
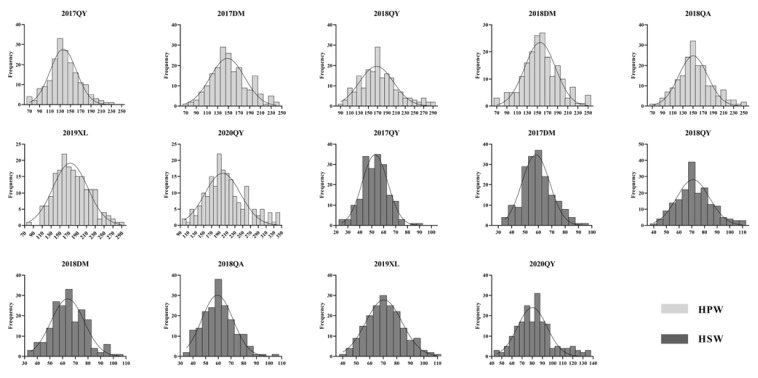
Frequency distribution of phenotype data of HPW and HSW in RIL population under different environments. Gray rectangle displays HPW. Black rectangle displays HSW. The *x*-axis indicates the values of HPW or HSW in seven environments (2017QY, 2017DM, 2018QY, 2018DM, 2018QA, 2019XL and 2020QY). The *y*-axis shows the number of individuals in the RIL population.

**Figure 3:**
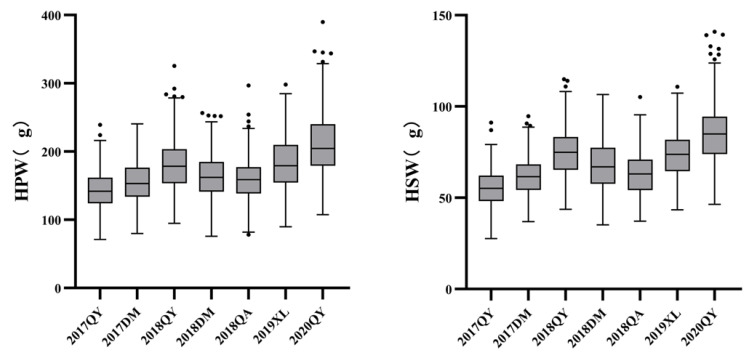
Box plots of HSW (**left**) and HPW (**right**) of RIL population under different environments.

In the two parents, “JH5” indicated greater HPW and HSW than “M130” in all seven environments, and the RIL population exhibited adequate variation types (Table 1). The distribution of HPW and HSW’s median and dispersion fluctuates marginally for each environment, showed a right-skewed pattern (Figure 2 and Figure 3). The phenotypic data of HPW and HSW were continuously distributed in the RIL population and confirmed to be normally distributed by the Shapiro–Wilk (*w*) test (Table 1 and Figure 2). The HPW and HSW of female parent JH5 varied from 213.2 to 291.31 g and 96.12 to 114.32 g, while the HPW and HSW of male parent M130 varied from 155.79 to 177.85 g and 63.88 to 76.2 g in the seven environments, respectively (Table 1). The HPW and HSW were significantly positively correlated in all seven environments, with a correlation coefficient range from 0.844 to 0.962 (Table 2). There were high phenotypic variations of HPW and HSW, with ranges of 71.04–239.22 g, 79.86–240.61 g; 94.85–325.53 g, 75.99–256.43 g; 78.28–296.87 g, 89.71–298.00 g; 107.61–389.97 g, 27.73–91.24 g; 36.93–94.67 g, 43.73–114.88 g; 35.13–106.51 g, 37.21–105.51 g; and 43.44–110.75 g, 46.39–140.89 g during 17QY, 17DM, 18QY, 18DM, 18QA, 19XL and 20QY, respectively. The broad-sense heritability of HPW and HSW were estimated to be 0.64 and 0.52. Analysis of variance (ANOVA) showed that genotype, environmental and genotype-by-environment interaction had a significant effect on HPW and HSW (Table 3).

### 3.2. Integrated Genetic Map Construction and Marker Distribution

**Figure 4:**
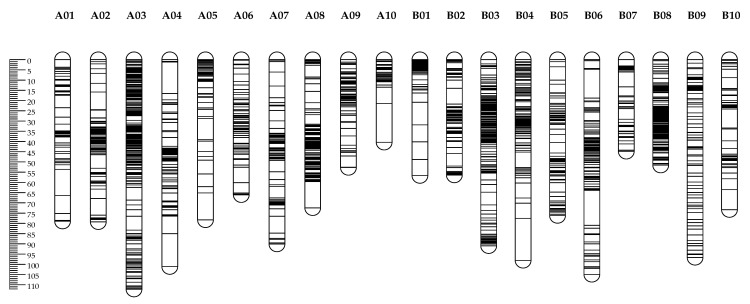
IHDGM of the RIL population. Left ruler was the length of linkage group. Black indicator displays position of each marker on IHDGM.

A total of 377 SSRs (9.51%), 131 AhTEs (14.15%), 90 SRAP primer pairs (37.81%) and 42 TRAP primer pairs (27.09%) had clear bands and excellent polymorphism between JH5 and M130. These polymorphism primers were used to obtain genotype data from the RIL population. In addition, 2808 SNP genotypic data from our previous study were also deployed to create an integrated high-density genetic linkage map (IHDGM) in the present study. Finally, an IHDGM with 3130 loci, covering 1998.92 cM with an average distance of 0.64 cM, was constructed on 20 chromosomes, including 2796 SNPs, 229 SSRs, 30 AhTEs, 56 SRAPs and 19 TRAPs. Of these, the “A” subgroup contained 1594 loci spanning 1038.87 cM with an average distance 0.68 cM, and the “B” subgroup contained 1536 loci spanning 960.05 cM with an average distance 0.63 cM. The length of a single linkage group ranged from 50.2 to 192 cM, and the maximum gap between markers was 20.58 cM (Table 4 and Table S1, Figure 4).

### 3.3. QTL Identification

**Figure 5:**
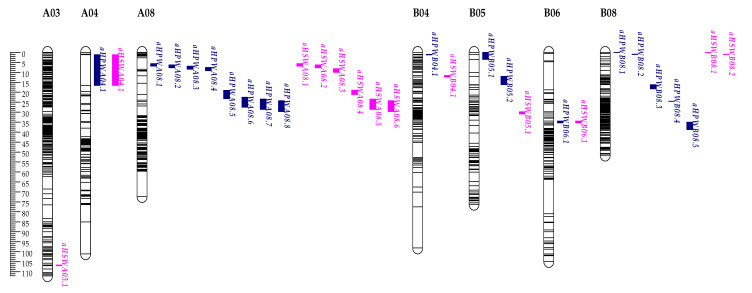
The distribution of QTLs for HPW (blue indicator) and HSW (pink indicator) on the genetic linkage map.

For HPW, 18 associated QTLs were identified in seven environments of four years, and distributed on chromosomes A04, A08, B04, B05, B06 and B08 (Table 5, Figure 5). These QTLs explained 3.662–10.826% of the phenotypic variation, with LOD values varying between 2.569 and 7.307. Among them, *qHPWA08.3* was repeatedly detected in three environments (17QY, 18DM and 19XL), and was located in the AhTE0658-TC22C01 interval of the A08 chromosome. The LOD values were 3.600, 7.307 and 5.436, and the PVE values were 4.407%, 8.893% and 6.788%, respectively. *qHPWA08.8* was repeatedly detected in two environments (18DM and 20QY), and was located in the Ah4-4-Ah2TC09B08 interval of the A08 chromosome. The LOD values were 4.602 and 3.291, and the PVE values were 3.731% and 5.316%, respectively. *qHPWB06.1* was repeatedly detected in two environments (17QY and 17DM). It was located on the SMK2106-SMK2107 interval of chromosome B06, with LOD values of 2.840 and 4.052, and PVE values of 5.993 and 8.481, respectively. *qHPWB08.1* was repeatedly detected in three environments (17DM, 18QA and 19XL), and was located in the AHGS1286-Ah3TC20B05 interval of the B08 chromosome. The LOD values were 5.473, 3.218 and 3.505, and the PVE values were 6.199%, 3.662% and 3.728%, respectively. *qHPWB08.2* was repeatedly detected in three environments (17QY, 18DM and 20QY), in AHGS1286-Ah3TC2 on chromosome B08. The LOD values were 3.972, 5.904 and 4.790, and the PVE values were 4.416, 6.574 and 5.108, respectively. Sixteen QTLs (61.5%) with positive additive effects (7.51–20.94) were contributed by the large-pod variety JH5 alleles.

For HSW, 13 QTLs linked with HSW were found in seven environments of four years (Table 5, Figure 5), which were distributed on chromosomes A03, A04, A08, B04, B05, B06 and B08. These QTLs explained 4.138–10.425% of the phenotypic variation, with LOD values varying between 2.545 and 6.662. Among them, *qHSWA03.1* was repeatedly detected in two environments (17DM and 17QY), located in the SMK539-SMK540 interval of the A03 chromosome, with LOD values of 4.039 and 2.671, and PVE values of 8.544 and 5.865, respectively. *qHSWA08.5* was repeatedly detected in two environments (18QY and 19XL), and was located in the Ah4-4-Ah2TC09B08 interval of the A08 chromosome. The LOD values were 6.662 and 5.739, and the PVE values were 9.197% and 6.594%, respectively. *qHSWA08.6* was repeatedly detected in three environments (17QY, 18DM and 20QY), and was located in the Ah4-4-Ah2TC09B08 interval of the A08 chromosome. The LOD values were 3.494, 3.491 and 3.996, and the PVE values were 4.868%, 4.849% and 4.627%, respectively. Nine QTLs (76.5%) with positive additive effects (2.62–6.31) were contributed by the large-seed variety JH5 alleles.

### 3.4. QTL Hotspot and Candidate Genes on A08

**Figure 6:**
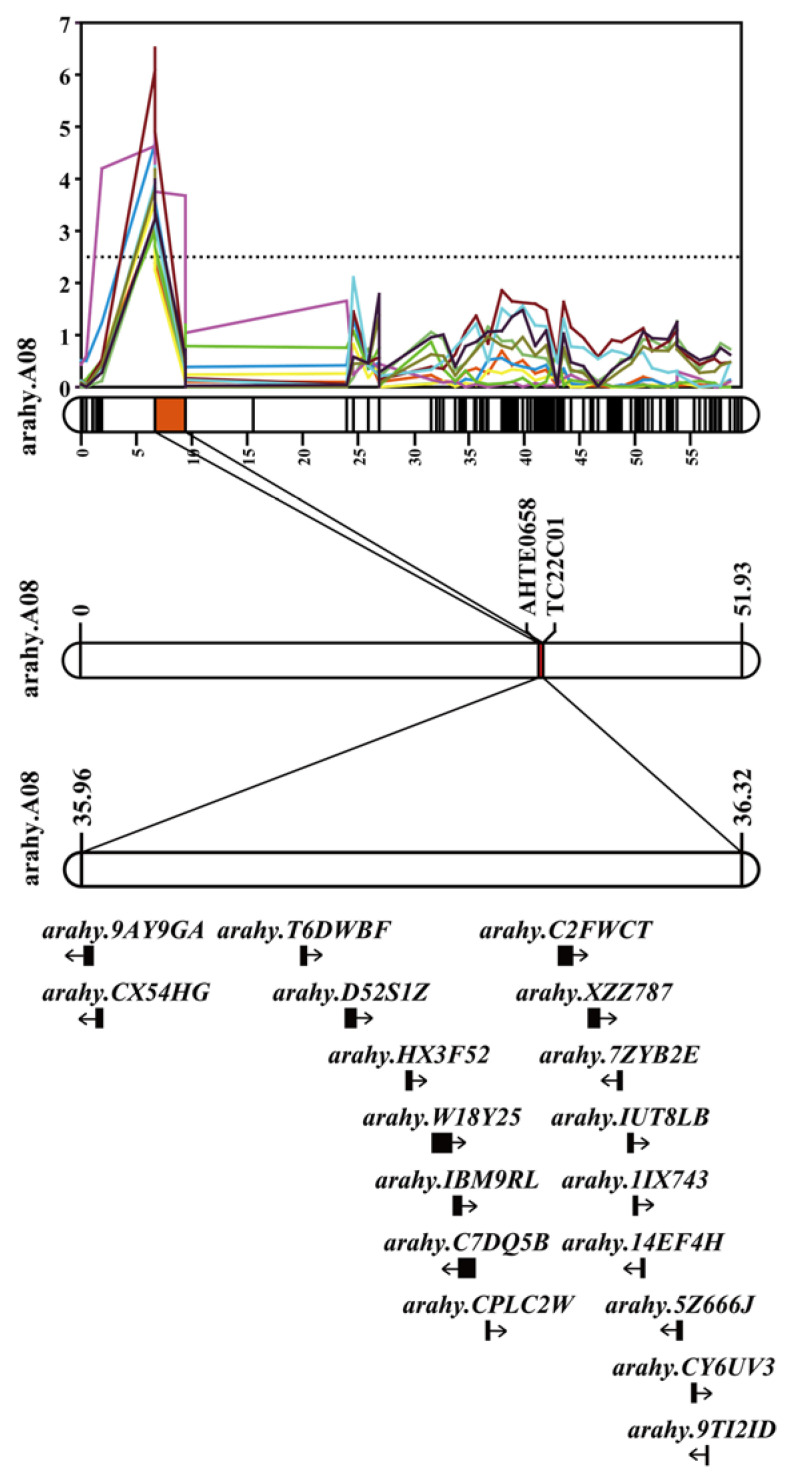
Candidate genes for the co-localization interval of HPW and HSW. Co-localization interval LOD mapped on A08. Candidate interval corresponds to the physical position of chromosome A08. Distribution of candidate genes in the co-localization interval on A08.

Based on multi-environment QTL co-localization analysis, high LOD intervals for HPW and HSW were detected in several conditions (Figure 6A). A total of 10 QTLs (HPW for 17QY, 17DM, 18DM, 18QA, 19XL and 20QY, HSW for 18DM, 18QA, 19XL and 20QY) associated with peanut traits of HPW and HSW on chromosome A08 were mapped using the flanking markers AhTE0658 and TC22C01 (Figure 6B), covering a genetic distance of 2.75 cM. The physical locations of markers AhTE0658 and TC22C01 are 35,963,966 bp and 36,328,872 bp, respectively, spanning a physical interval of 0.36 Mb on chromosome A08.

The QTL hotspot interval on A08 was mapped on 6.67–9.42 cM in this map. This interval was mapped at 35,963,966–36,328,872 bp in chromosome A08 by the flanking markers AhTE0658 and TC22C01 (Figure 6C). The 0.36 Mb interval contained 18 putative genes (https://legacy.peanutbase.org/gb2/gbrowse/arahy.Tifrunner.gnm2/, accessed on 15 August 2023). Of these, annotation information of 17 genes had been described, and one gene was described as an unknown protein (Table 6). *Arahy.W18Y25* and *Arahy.CPLC2W* encode a PPR superfamily and a PPR-like superfamily, respectively. *Arahy.IBM9RL, Arahy.14EF4H* and *Arahy.D52S1Z* encode FERTILIZATION-INDEPENDENT ENDOSPERM-like (FIE), sugar transporter 11 and 2-oxoglutarate/Fe(II)-dependent dioxygenase-like, respectively. Twelve of eighteen genes were assigned at least one GO term. These 18 genes are divided into three GO categories, cellular components with 3 genes, molecular functions with 11 genes and biological processes with 9 genes. Enrichment analysis indicated that four candidate genes were enriched, including translational initiation, cell redox homeostasis, extrinsic component of membrane, transferase activity and transferring glycosyl groups (Table S2). Through KEGG enrichment analyses, four genes were found to be involved with fatty acid elongation, diterpenoid biosynthesis, photosynthesis and fatty acid metabolism biosynthesis (Table S3).

## 4. Discussion

The completion of whole genome sequencing for the tetraploid peanut and whole genome resequencing of several cultivated varieties have minimized the likelihood of marker position discrepancies, consequently enhancing the precision of QTL/gene mapping [20,45]. SNP, as a third-generation molecular marker, compared with the previous two generations, showed a more abundant polymorphism in peanut germplasm resources. Constructing a high-density genetic map through developing molecular markers led to improved efficiency and accuracy of QTL mapping of interest traits. So far, many studies have identified QTLs using genetic linkage maps of different molecular markers in the peanut [24,37,46,47,48]. The density of the genetic map increased from hundreds to thousands. However, the most high density genetic maps only contained 1 to 2 molecular marker types. For example, our previous study constructed a HDGM with 2808 SNPs [25], and another HDGM with 2996 SNPs and 330 SSRs [27]. Hu et al. [26] constructed a HDGM with 68 SSRs and 2266 SNPs. To further improve the accuracy and consistency of QTL detection, we constructed an integrated high-density genetic map using five types of molecular markers in this study. This map contained 3130 loci using 2796 SNPs, 229 SSRs, 30 AhTEs, 56 SRAPs and 19 TRAPs, and is currently the most comprehensive and integrated record of marker data available. Despite our efforts, we were not able to confirm the localization markers consistent with prior published results. This could be attributed to different mapping populations or the absence of adequate map density. As a result, we are compelled to consider the necessity of further refining our integrated map.

Pod and seed weight are crucial indicators of yield and have been extensively studied in various crops [49,50,51]. However, the genetic mechanism underlying these traits in peanut seeds remains unclear and requires further investigation. Up to the present date, several QTLs related to pod and seed traits have been discovered on different chromosomes in peanuts [52,53]. In recent years, QTLs for pod and seed weight identified on A05, A07 and B06 have been repeatedly reported [34,54,55]. Similarly, in our study, QTLs for HPW and HSW were identified on B06, except for A05 and A07. Notably, *qHPWA08.3*, *qHPWB08.1*, *qHPWB08.2* and *qHSWA08.6* were identified in more than three environments, suggesting the stability of their genetic effects across different conditions. In addition, two QTL clusters for HPW were found on A08. It is worth noting that *qHPWA08.1*, *qHPWA08.2*, *qHPWA08.3*, *qHPWA08.4*, *qHSWA08.1*, *qHSWA08.2* and *qHSWA08.3* were located in the 0.36 Mb genome interval, but each QTL showed a micro effect and less than 10% PVE. QTL clusters could be considered multifactorial linkages, in accordance with the polygene hypothesis, and when consistently detected across multiple environments, they show a stronger association with traits [56]. Consequently, QTL clusters on A08 in the current study, which were detected repeatedly in multiple environments, were considered to be strongly correlated with pod and seed weight. Our findings offer valuable insights into the genetic basis of pod and seed weight traits in the peanut, and the suggestion that the QTL cluster related HPW and HSW on A08 enhances our confidence in the accuracy of the QTLs identified on other chromosomes.

In this study, we identified 18 potential genes situated on A08, covering a physical interval of 0.36 Mb. Of these, there were five candidate genes that might regulate seed development in the plant. For instance, 2-oxoglutarate/Fe(II)-dependent dioxygenase-like possesses intrinsic functions in DNA repair, epigenetics, and post-translational modification, as well as the activation and catabolism of plant growth regulators. Furthermore, it orchestrates the production and catabolism of numerous plant hormones, including gibberellins (GAs), ethylene, auxin (IAA) and salicylic acid (SA) [57]. The FIE protein serves as a vital structural component of expected PRC2 complexes, and plays a critical role in various plant growth stages, such as seed development, the transition from the vegetative phase, and the response to vernalization [58]. The PPR family of proteins is an important family of genes involved in a multitude of plant growth and development processes. PPR proteins, which bind to RNA, participate in various post-transcriptional regulatory processes, and play pivotal roles in the development of plant leaves, seed development and response to stress [59]. Sugar transporter proteins play a crucial role in the maturation of cereal crops. They provide possible genetic pathways for enhancing seed filling and productivity, particularly in maize and other grain crops [60,61]. In this study, genes *Arahy.W18Y25* and *Arahy.CPLC2W* encoded the PPR and PPR-like proteins superfamilies, respectively. *Arahy.IBM9RL*, *Arahy.14EF4H* and *Arahy.D52S1Z* encoded FIE, sugar transporter 11 and 2-oxoglutarate/Fe(II)-dependent dioxygenase-like, respectively. Therefore, we suggested that these candidate genes may participate in regulating seed development, such as seed size and weight, and speculated that the QTL hotspot (35,963,966–36,328,872 bp) on A08 was also a vital genome region for pod and seed weight.

## 5. Conclusions

In the present study, a RIL population was constructed using female parent JH5 and male parent M130. A HDGM was constructed including a total of 3130 marker loci and spanning a 1998.92 cM genetic distance. In total, 31 QTLs for HPW and HSW were detected on A03, A04, A08, B04, B05, B06 and B08. A QTL hotspot was identified on A08, which was across a 0.36 Mb physical interval and included 18 candidate genes. This work will provide favorable information for researchers to breed high-yield cultivars and an analysis of the genetic mechanisms for pod and seed weight in the peanut.

## Figures and Tables

**Table 1:** Descriptive statistics of HPW and HSW for parents and RIL populations.

Traits	Env.^a^	Parents		RIL Population							
		JH5	M130	Min^b^	Max^c^	Mean	SD	CV (%)	Shapiro–Wilk (*w*-Test)	Skew^d^	Kurt^e^
HPW(g)	17QY	254.03 ± 6.84 **	170.13 ± 0.91	71.04	239.22	143.55	30.37	21.16	0.99	0.23	0.21
	17DM	247.18 ± 2.30 **	164.64 ± 3.78	79.86	240.61	156.36	33.06	21.14	0.99 *	0.36	−0.11
	18QY	271.28 ± 8.80 **	184.48 ± 2.45	94.85	325.53	179.42	42.91	23.91	0.98 **	0.62	0.36
	18DM	236.77 ± 6.46 **	155.79 ± 3.57	75.99	256.43	162.68	35.68	21.93	0.99	0.18	0.09
	18QA	233.38 ± 1.89 **	159.70 ± 5.95	78.28	296.87	159.36	35.14	22.05	0.98 *	0.51	0.86
	19XL	213.20 ± 1.74 **	167.76 ± 10.46	89.71	298.00	183.19	38.75	21.15	0.99	0.39	−0.17
	20QY	291.31 ± 1.41 **	177.85 ± 0.92	107.61	389.97	212.66	52.48	24.68	0.97 **	0.66	0.36
HSW(g)	17QY	105.22 ± 2.17 **	70.04 ± 0.69	27.73	91.24	55.42	11.17	20.16	0.99	0.19	0.30
	17DM	100.82 ± 1.49 **	68.83 ± 1.14	36.93	94.67	62.00	11.60	18.71	0.99	0.32	−0.10
	18QY	114.32 ± 2.73 **	76.20 ± 3.11	43.73	114.88	74.99	14.47	19.29	0.99	0.33	−0.14
	18DM	96.12 ± 2.48 **	63.88 ± 1.06	35.13	106.51	67.30	13.70	20.35	0.99	0.25	−0.06
	18QA	100.24 ± 1.44 **	63.90 ± 1.65	37.21	105.15	63.18	13.04	20.64	0.98	0.39	−0.05
	19XL	109.07 ± 0.61 **	74.07 ± 2.70	43.44	110.75	73.99	13.40	18.10	1.00	0.26	−0.14
	20QY	109.57 ± 1.05 **	71.01 ± 1.34	46.39	140.89	85.87	18.73	21.81	0.96 **	0.68	0.53

* and ** showed significant differences at the levels of 0.05 and 0.01, respectively. Env.^a^—environments; 17DM, 17QY, 18DM, 18QY, 18QA, 19XL and 20QY represent sampling from 2017 to 2020 at Daming (DM), Qingyuan (QY), Qian’an (QA) and Xinle (XL); Min^b^—minimum value of different environments; Max^c^—maximum value of different environments; Skew^d^—skewness; Kurt^e^—kurtosis.

**Table 2:** Simple correlation coefficients between HPW and HSW under seven environments.

Env.	Traits	HPW	HSW
17QY	HPW	1	0.844 **
	HSW	0.844 **	1
17DM	HPW	1	0.848 **
	HSW	0.848 **	1
18QY	HPW	1	0.881 **
	HSW	0.881 **	1
18DM	HPW	1	0.908 **
	HSW	0.908 **	1
18QA	HPW	1	0.858 **
	HSW	0.858 **	1
19XL	HPW	1	0.881 **
	HSW	0.881 **	1
20QY	HPW	1	0.962 **
	HSW	0.962 **	1

**, significant correlation at 0.01 level.

**Table 3:** Results of ANVOA and *h_B_*^2^ of HPW and HSW.

Traits	Variables	*df*	MS	F-Value	*p*-Value	*h_B_* ^2^
HPW	Geno.	187	20956.938	314.2	*p* < 0.001	0.64
	Env.	6	289675.957	4343.012	*p* < 0.001	
	G × E	1122	1828.708	27.417	*p* < 0.001	
HSW	Geno.	187	2566.214	182.797	*p* < 0.001	0.52
	Env.	6	56659.777	4035.995	*p* < 0.001	
	G × E	1122	249.054	17.741	*p* < 0.001	

Geno., Env. and G × E are abbreviation of genotype, environment and G × E interaction.

**Table 4:** Information of IHDGM.

Linkage Groups	No. of Markers	Length of Linkage Group (cM)	Average Distance (cM)	Maximum Gap (cM)
A01	65	110.60	1.70	12.85
A02	256	92.49	0.36	8.18
A03	259	104.09	0.40	6.76
A04	99	105.20	1.06	16.03
A05	238	131.83	0.55	13.20
A06	264	72.80	0.28	7.51
A07	119	182.98	1.54	8.14
A08	159	117.05	0.74	12.74
A09	91	63.74	0.70	5.39
A10	44	58.09	1.32	18.93
A subgroup	1594	1038.87	0.68	
B01	203	50.20	0.25	11.12
B02	84	90.38	1.08	6.21
B03	153	76.53	0.50	6.21
B04	196	65.14	0.33	20.59
B05	96	72.62	0.76	6.58
B06	247	192.61	0.78	17.14
B07	48	59.51	1.24	7.25
B08	313	138.82	0.44	2.23
B09	104	54.89	0.53	3.63
B10	92	159.35	1.73	9.87
B subgroup	1536	960.05	0.63	
Whole genome	3130	1998.92	0.64	

**Table 5:** QTL mapping results of HPW and HSW.

Traits	QTLs	Env.^a^	Chr.^b^	Position (cM)	Marker Interval	LOD	PVE (%)	Add^c^	Dir^d^
HPW	*qHPWA04.1*	18QY	A04	2.21	SMK547-SMK549	3.42	8.11	12.27	JH5
	*qHPWA08.1*	20QY	A08	6.26	AhTE0658-TC22C01	6.69	8.07	16.38	JH5
	*qHPWA08.2*	17DM	A08	6.97	AhTE0658-TC22C01	3.38	4.55	7.93	JH5
	*qHPWA08.3*	17QY	A08	7.69	AhTE0658-TC22C01	3.60	4.41	7.64	JH5
		18DM	A08	7.69	AhTE0658-TC22C01	7.31	8.89	11.85	JH5
		19XL	A08	7.69	AhTE0658-TC22C01	5.44	6.79	11.23	JH5
	*qHPWA08.4*	18QA	A08	8.40	AhTE0658-TC22C01	6.07	7.83	10.34	JH5
	*qHPWA08.5*	18QY	A08	21.20	me3em14-196-Ah4-4	4.32	6.21	12.02	JH5
	*qHPWA08.6*	19XL	A08	25.00	Ah4-4-Ah2TC09B08	4.92	5.54	10.09	JH5
	*qHPWA08.7*	18QA	A08	26.00	Ah4-4-Ah2TC09B08	3.60	4.55	7.85	JH5
	*qHPWA08.8*	18DM	A08	27.00	Ah4-4-Ah2TC09B08	4.60	5.32	9.13	JH5
		20QY	A08	27.00	Ah4-4-Ah2TC09B08	3.29	3.73	11.08	JH5
	*qHPWB04.1*	17QY	B04	1.11	SMK1996-SMK1995	3.20	6.77	−9.71	M130
	*qHPWB05.1*	19XL	B05	3.01	SMK2087-SMK2088	2.60	5.72	−10.30	M130
	*qHPWB05.2*	19XL	B05	16.01	SMK2085-SMK2084	3.19	6.70	−13.45	M130
	*qHPWB06.1*	17QY	B06	34.51	SMK2106-SMK2107	2.84	5.99	7.51	JH5
		17DM	B06	34.51	SMK2106-SMK2107	4.05	8.48	9.92	JH5
	*qHPWB08.1*	17DM	B08	0.00	AHGS1286-Ah3TC20B05	5.47	6.20	−9.30	M130
		18QA	B08	0.00	AHGS1286-Ah3TC20B05	3.22	3.66	−7.07	M130
		19XL	B08	0.00	AHGS1286-Ah3TC20B05	3.51	3.73	−8.34	M130
	*qHPWB08.2*	17QY	B08	1.00	AHGS1286-Ah3TC20B05	3.97	4.42	−7.66	M130
		18DM	B08	1.00	AHGS1286-Ah3TC20B05	5.90	6.57	−10.21	M130
		20QY	B08	1.00	AHGS1286-Ah3TC20B05	4.79	5.11	−13.05	M130
	*qHPWB08.3*	20QY	B08	17.21	SMK2658-SMK2393	2.65	10.83	20.94	JH5
	*qHPWB08.4*	20QY	B08	24.51	SMK2406-SMK2423	2.57	5.56	16.27	JH5
	*qHPWB08.5*	18QA	B08	36.81	SMK2628-SMK2626	3.65	8.09	−12.85	M130
HSW	*qHSWA03.1*	17DM	A03	106.81	SMK539-SMK540	4.04	8.54	−3.88	M130
		18QY	A03	106.81	SMK539-SMK540	2.67	5.87	−3.74	M130
	*qHSWA04.1*	17DM	A04	1.21	SMK547-SMK549	2.75	5.79	2.84	JH5
	*qHSWA08.1*	20QY	A08	6.23	AhTE0658-TC22C01	5.65	6.93	5.32	JH5
	*qHSWA08.2*	19XL	A08	6.97	AhTE0658-TC22C01	3.75	4.47	3.30	JH5
	*qHSWA08.3*	18DM	A08	9.12	AhTE0658-TC22C01	5.00	6.00	3.52	JH5
	*qHSWA08.4*	18QA	A08	19.00	Ah1TC06H03-AhTE0477	3.91	6.37	3.59	JH5
	*qHSWA08.5*	18QY	A08	26.00	Ah4-4-Ah2TC09B08	6.66	9.20	4.52	JH5
		19XL	A08	26.00	Ah4-4-Ah2TC09B08	5.74	6.59	3.99	JH5
	*qHSWA08.6*	17QY	A08	27.00	Ah4-4-Ah2TC09B08	3.49	4.87	2.62	JH5
		18DM	A08	27.00	Ah4-4-Ah2TC09B08	3.49	4.85	3.16	JH5
		20QY	A08	27.00	Ah4-4-Ah2TC09B08	4.00	4.63	4.32	JH5
	*qHSWB04.1*	18QA	B04	11.61	SMK1978-SMK1848	4.39	10.43	5.84	JH5
	*qHSWB05.1*	20QY	B05	29.91	SMK2063-SMK2062	2.55	5.61	6.31	JH5
	*qHSWB06.1*	17QY	B06	34.51	SMK2106-SMK2107	3.89	8.52	3.26	JH5
	*qHSWB08.1*	18DM	B08	0.00	AHGS1286-Ah3TC20B08	4.73	5.50	−3.38	M130
	*qHSWB08.2*	20QY	B08	1.00	AHGS1286-Ah3TC20B08	3.86	4.14	−4.11	M130

Env.^a^—environment; Chr.^b^—chromosome; Add^c^—additive effect; Dir^d^—direction.

**Table 6:** Gene annotation in candidate regions.

Chr.	Gene Names	Physical Position (bp)	Nr_Annotation
A08	*Arahy.9AY9GA*	35,966,338~35,970,068	DDRGK domain-containing protein 1-like
A08	*Arahy.CX54HG*	35,973,257~35,975,499	Translation initiation factor SUI1 family protein
A08	*Arahy.T6DWBF*	36,090,499~36,092,669	Trafficking protein particle complex subunit-like protein
A08	*Arahy.D52S1Z*	36,116,001~36,121,083	Probable 2-oxoglutarate/Fe(II)-dependent dioxygenase-like
A08	*Arahy.HX3F52*	36,151,497~36,153,080	Calcium-dependent lipid-binding family protein
A08	*Arahy.IBM9RL*	36,178,370~36,181,596	Polycomb group protein fertilization-independent endosperm-like (FIE)
A08	*Arahy.W18Y25*	36,166,807~36,175,891	Pentatricopeptide repeat (PPR) superfamily protein
A08	*Arahy.C7DQ5B*	36,181,753~36,189,459	Unknown protein
A08	*Arahy.CPLC2W*	36,196,903~36,197,724	Pentatricopeptide repeat (PPR-like) superfamily protein
A08	*Arahy.C2FWCT*	36,238,299~36,245,344	Breast carcinoma amplified sequence 3 protein
A08	*Arahy.XZZ787*	36,255,496~36,260,840	Probable galacturonosyltransferase 12-like
A08	*Arahy.7ZYB2E*	36,272,312~36,273,769	Thioredoxin 2
A08	*Arahy.IUT8LB*	36,278,268~36,280,173	Oxygen-evolving enhancer protein
A08	*Arahy.1IX743*	36,281,314~36,282,631	Papain family cysteine protease
A08	*Arahy.14EF4H*	36,285,790~36,286,678	Sugar transporter 11
A08	*Arahy.CY6UV3*	36,314,963~36,316,298	Papain family cysteine protease
A08	*Arahy.5Z666J*	36,306,402~36,308,262	Unknown protein
A08	*Arahy.9TI2ID*	36,317,379~36,323,108	Papain family cysteine protease

Chr. chromosome.

## Data Availability

The data generated and analyzed from this study has been incorporated into the manuscript and supplementary files.

## Supplementary Materials

The following supporting information can be downloaded at: https://www.mdpi.com/article/10.3390/genes14091792/s1, Table S1—Marker position information of the integrated high-density genetic map. Table S2—GO annotation of candidate regions. Table S3—KEGG annotation of candidate regions.
